# Large cell neuroendocrine carcinoma of the cervix: a case report

**DOI:** 10.3389/fonc.2024.1419710

**Published:** 2024-07-24

**Authors:** Chunmei Li, Maoyuan Wu, Wenwen Zhang, Xiaoling Jiang, Lixia Zhang, Gangcheng Wang, Lianli He

**Affiliations:** ^1^ Department of Gynecology, The Third Affiliated Hospital of Zunyi Medical University (The First People’s Hospital of Zunyi), Zunyi, Guizhou, China; ^2^ Department of Abdominal and Pelvic Tumor Surgery, The First Affiliated Hospital of Zhengzhou University, Zhengzhou, Henan, China

**Keywords:** case report, advanced cervical cancer, large cell, cervical cancer treatment, well-differentiated neuroendocrine carcinoma

## Abstract

Large Cell Neuroendocrine Carcinoma (LCNEC) of the cervix is an extremely rare but highly aggressive type of cervical cancer and it requires multimodal therapy to improve their quality of life. At present, there are no established, standardized treatment protocols for managing large cell neuroendocrine carcinoma of the cervix. In this report, we present a case of a patient with cervical LCNEC, Who was a 39-year-old woman who presented with irregular vaginal bleeding accompanied by lower abdominal distension for over a month. Examination revealed a cauliflower-like cervical mass approximately 4cm in diameter, with the normal cervical architecture distorted and partially fused to the vaginal wall. Following further investigations, the stage assigned was IVB, and who was started on neoadjuvant chemotherapy with the TC (paclitaxel + carboplatin) regimen but during neoadjuvant chemotherapy, The patient developed a vaginal urinary leakage. Then, The patient underwent a comprehensive treatment regimen that included pelvic exenteration, urinary system reconstruction, pelvic floor reconstruction, and chemotherapy. Given the patient’s positive immunohistochemistry for EGFR, the treatment was combined with the anti-angiogenic drug, bevacizumab. The patient achieved complete remission following the comprehensive treatment. Through this case to explore individualized treatment for cervical LCNEC.

## 1 Introduction

Currently, there is no standardized treatment protocol for large cell neuroendocrine carcinoma of the cervix. Treatment approaches often reference those used for common cervical cancer, small cell neuroendocrine carcinoma of the cervix, and small cell neuroendocrine carcinoma of the lung due to its rarity and aggressive nature, which generally leads to poor prognosis. Recent studies have found that the prognosis of patients with cervical cancer may be improved by combining immunotherapy and targeted therapy with traditional radiotherapy and chemotherapy, thereby enhancing their quality of life. This report presents a case of advanced large cell neuroendocrine carcinoma of the cervix to explore personalized treatment options for advanced stages of this special type of cervical cancer.

## 2 Case report

**Figure 1:**
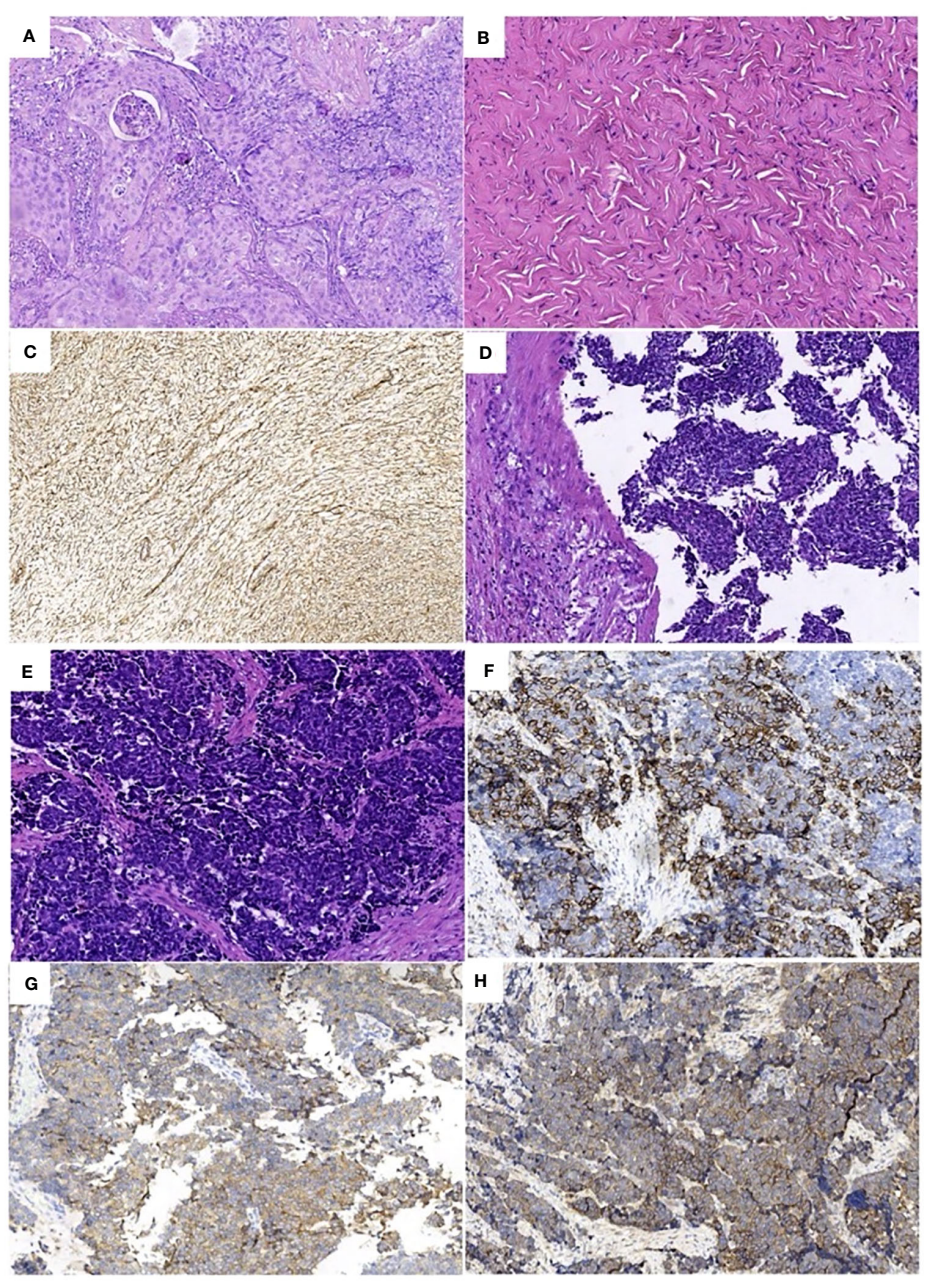
Pathologic findings: **(A)** Non-keratinizing squamous cell carcinoma of the cervix (HE×200), **(B)** Right ovarian fibroma (HE×200), Immunohistochemistry: **(C)** (Vimentin ×200) positive. **(D)**. **(E)** Large cell neuroendocrine carcinoma of the cervix (HE×200), Immunohistochemistry: **(F)** (CD56×200) positive, **(G)** (SYN×200) partially positive. **(H)** (EGFR ×200) positive.

**Figure 2:**
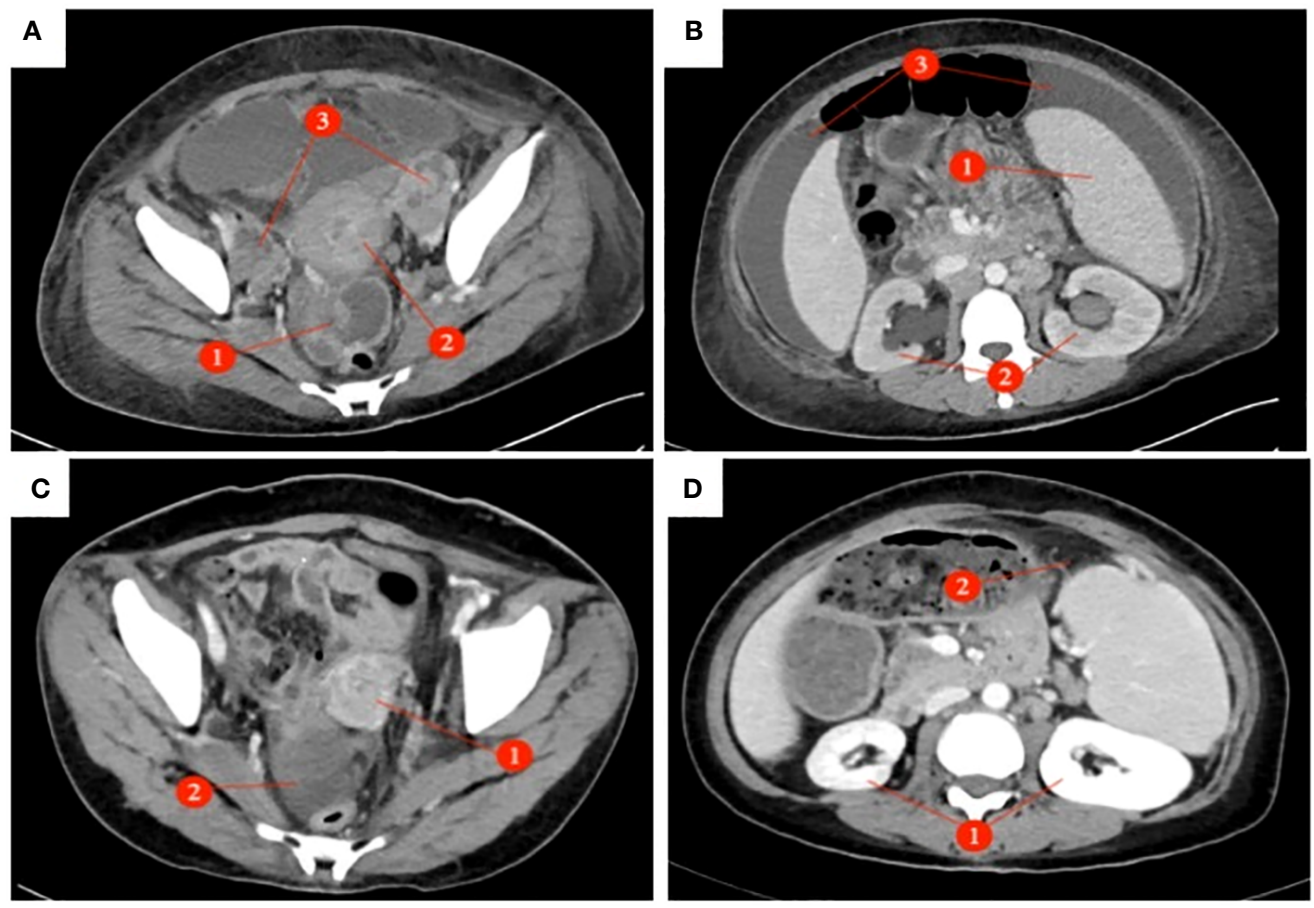
Abdominal and pelvic enhanced CT: (Before chemotherapy AB After chemotherapy CD) : **(A)** The pelvic mass presents as an irregular solid cystic lesion measuring 5×6cm, exhibiting patchy shadowing and demonstrating enhancement of both the solid component and cyst wall. (①); The cervix exhibited a 5 x 4cm mass with heterogeneous enhancement of soft tissue density. (õ②); Bilateral ovarian enlargement (③), Pelvic effusion. **(B)** Splenomegaly (①); bilateral hydronephrosis with bilateral hydrourete (②), Abdominal effusion (③). **(C)** Disappearance of cervical lesion (①), In the right adnexal area, the density shadow of irregular soft tissue mass was 4.1 x 4.3cm (②). **(D)** Disappearance of bilateral hydronephrosis (①), Splenomegaly, Splenic abscess (5.5×1.7cm) (②).

**Figure 3:**
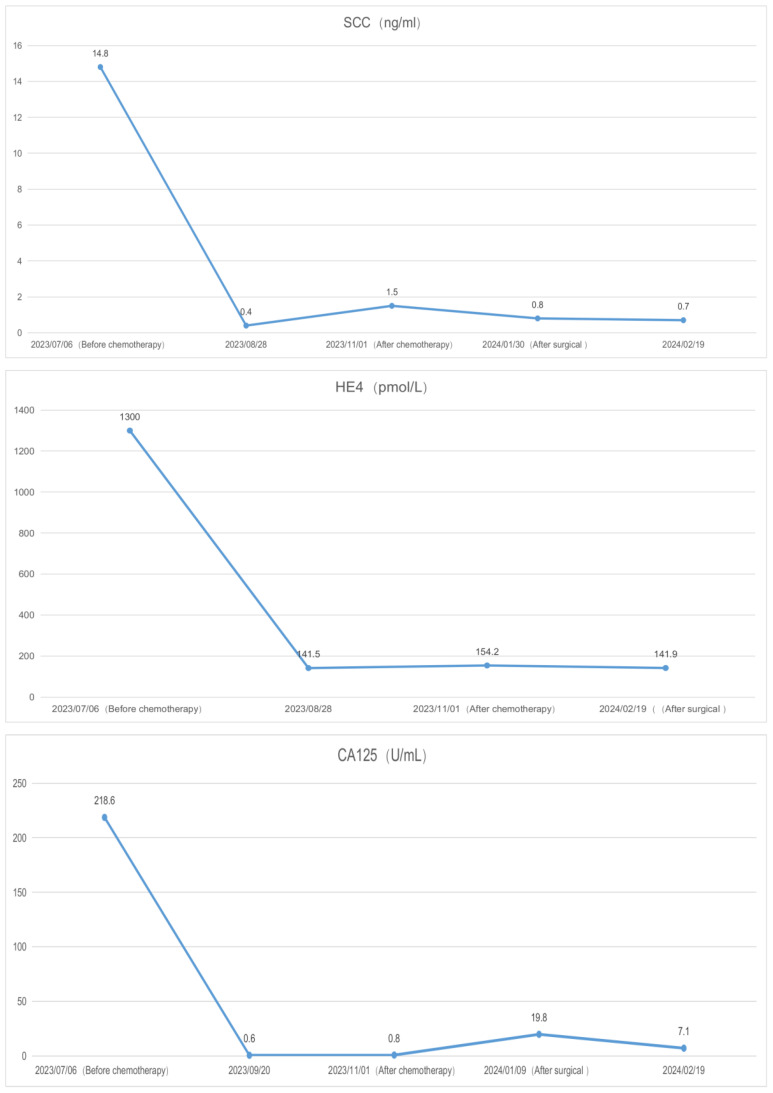
Tumor markers.

A 39-year-old female patient was admitted to the hospital due to “irregular vaginal bleeding accompanied by lower abdominal distension for over a month, worsening for the past 10 days.” Her menstrual cycle was regular, with a history of 1 cesarean section and 2 artificial abortions; she denied any family history of genetic disorders or cancer. Physical examination revealed a cauliflower-like cervical mass approximately 4cm in diameter, with the normal cervical architecture distorted and partially fused to the vaginal wall. Bleeding was noted upon contact. The vaginal fornices were obliterated, the vaginal length was markedly shortened to about 2cm, and nodular changes were present along the anterior vaginal wall, while the posterior vaginal wall appeared uninvolved; bimanual examination: the anterior vaginal wall showed nodular changes, was immobile, without elasticity, the posterior vaginal wall had acceptable elasticity, without palpable masses, the uterus was immobile and non-tender; the right adnexal area was thickened, immobile, without significant tenderness, the left adnexal area was non-tender, immobile, without palpable abnormalities. Trimanual examination: bilateral sacrouterine ligaments were thickened and shortened. Cervical biopsy: Non-keratinizing squamous cell carcinoma of the cervix (**Figure 1A**). Abdominal and pelvic contrast-enhanced CT revealed a cervical mass involving the upper vagina and adjacent bladder, with cystic and solid components suggestive of pelvic implantation metastases. Bilateral ovarian enlargement was also noted, raising concern for metastatic disease. (**Figure 2A**); abdominal and pelvic effusion, splenomegaly, bilateral hydronephrosis with bilateral hydroureter (**Figure 2B**); pelvic and left inguinal lymph node metastasis. Tumor markers (**Figure 3** before chemotherapy). Multiple paracentesis examinations did not reveal cancer cells.

**Figure 4:**
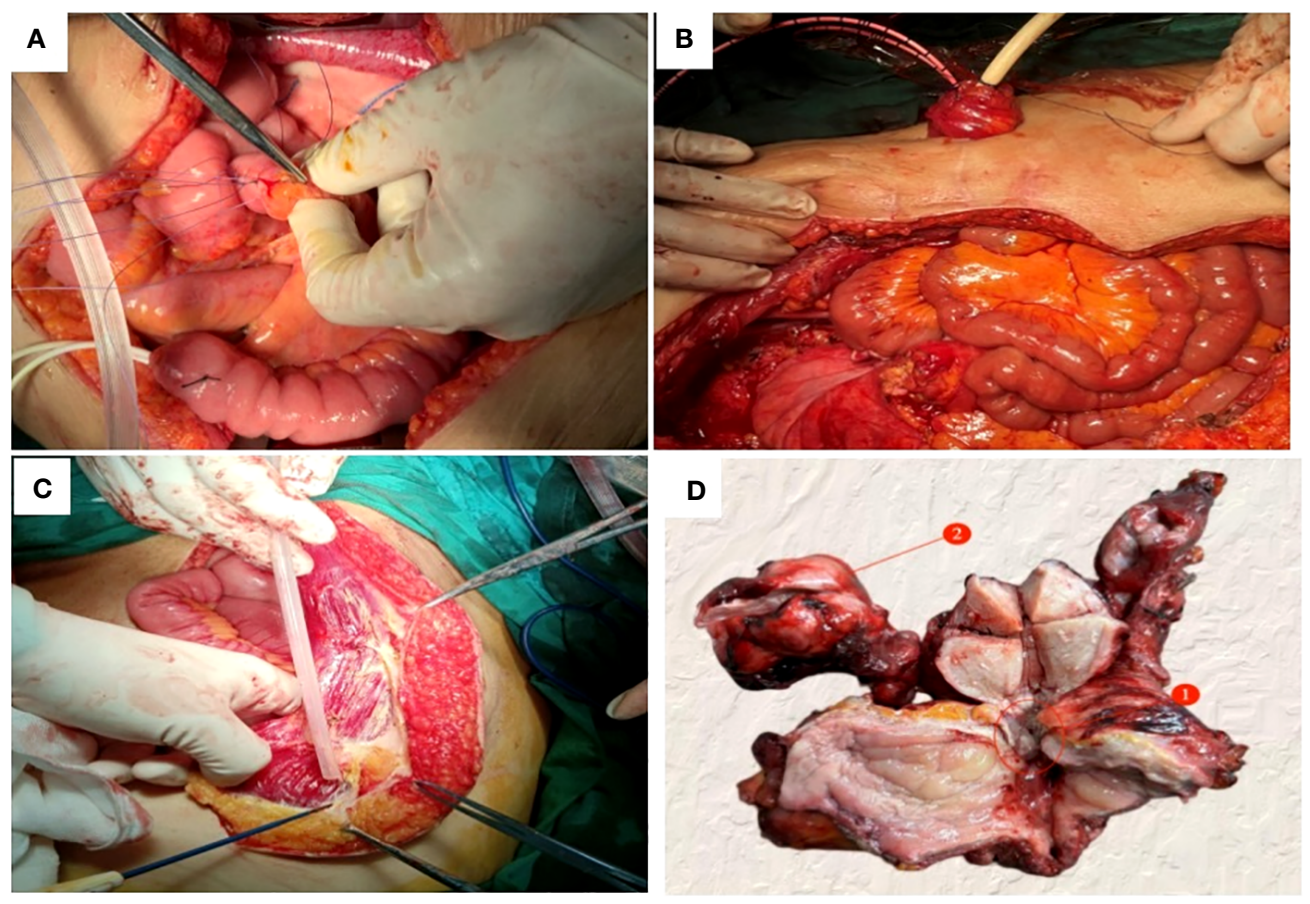
Pelvic exenteration was performed to reconstruct organ functions: **(A)** The proximal segment of the severed ileum was embedded and sutured, **(B)** The ileocystostomy was fixed, **(C)** Detach the right rectus muscle flap. **(D)** A fistula with a diameter of 0.5cm was found in the trigone of the bladder (①); The right ovary was enlarged about 4cm in diameter (②).

The preliminary diagnoses were: 1. Squamous cell carcinoma of the cervix, stage IVB. 2. Suspected ovarian cancer. After carefully evaluating and ruling out any contraindications, the patient was initiated on neoadjuvant chemotherapy with the TC (paclitaxel + carboplatin) regimen. Prior to the third cycle of chemotherapy, the patient developed a vesicovaginal fistula resulting in urinary leakage (**Figure 4D**). After four cycles of chemotherapy, a follow-up enhanced CT scan of the pelvis and abdomen showed a significant reduction in the size of the cervical lesion, which was indistinct, a reduction in the left adnexal lesion, which was unclear, and a slight reduction in the right adnexal lesion (**Figure 2C**); a decrease in ascites; the disappearance of bilateral hydronephrosis; splenomegaly, with possible splenic abscess (**Figure 2D**), and a reduction in pelvic and abdominal lymph nodes. Tumor markers were reassessed (**Figure 3** after chemotherapy).

Due to the patient’s inability to tolerate vaginal urinary leakage, and with her full informed consent and after excluding surgical contraindications, the surgical treatment had been performed. Intraoperatively, the cervical lesion was confirmed to have invaded the bladder, urethra, and upper vaginal segment. The right ovary was markedly enlarged, with an irregular surface and diameter of approximately 4cm, while no obvious abnormalities were noted in the left ovary or fallopian tubes. Total hysterectomy, bilateral adnexectomy, bladder, vagina, and urethra were removed. The spleen was explored, enlarged to about 20×9cm, with no cancer invasion observed, perisplenic abscess was present, pus was cleared, and the spleen was preserved. Resection included a 1.0cm tumor on the abdominal wall surface, a 1.5cm tumor on the surface of the descending colon, a 2.0cm tumor on the surface of the sigmoid colon, and other visibly affected areas. No obvious abnormalities were seen in the remainder of the pelvic and abdominal cavity. For urinary system reconstruction, approximately 25cm of the ileum, 30cm away from the ileocecal part, was selected to form a urinary pouch. The proximal end of the pouch was closed, and the distal end was brought out to the abdominal wall to form a stoma. The bilateral ureters were implanted into individual double-J stents, which were then anastomosed to the ileal conduit to allow for external drainage through the conduit stoma. The stent tubes were fixed to the ureters, the distal end of the ileal pouch, and the skin to prevent dislocation (**Figures 4A, B**). To reconstruct the pelvic floor, the anterior and posterior sheaths of the right rectus abdominis were separated from the rectus abdominis adhesions, preserving both sheaths. The upper edge of the rectus abdominis was detached 3cm below the right costal margin, and the rectus abdominis tendon was detached from the pubic crest, retaining only the right abdominal wall arteries and veins. The freed pedicled rectus abdominis muscle was pulled down to the pelvic floor, closing the vaginal stump to promote healing of the vaginal remnant, strengthen the anterior rectal wall to prevent fistula, eliminate pelvic floor space, reduce pelvic effusion, and prevent infection (**Figure 4C**). Postoperative examination of the specimen revealed visible tumor lesions near the urethra close to the bladder and on the posterior wall of the bladder, with a leak point near the anterior urethra in the bladder trigone area (**Figure 4D**). Postoperative pathology confirmed: 1. Large cell neuroendocrine carcinoma of the cervix and right ovary (**Figure 1E**), infiltrating the full thickness of the cervix, with vascular and nerve invasion; (cervix and cervical canal, posterior vaginal fornix, part of the uterine body) showed high-grade squamous intraepithelial lesion (HSIL/CIN III) involving glands; margins were tumor-free: cancer nodules found in the perivesical fibrofatty tissue (3 nodules): Immunohistochemistry: CD56 (+) (**Figure 1F**), Syn (partly +) (**Figure 1G**), P16 (+), EGFR (+) (**Figure 1H**), TTF-1 (-), CK5/6 (-), Ki-67 (+, about 70%); 2. Right ovarian fibroma (**Figure 1B**): Vimentin (+) (**Figure 1C**), Inhibin-a (-); 3. Eight left pelvic lymph nodes, four of which showed cancer metastasis (4/8), with no tumor tissue found in the remaining examined tissues.

The patient with large cell neuroendocrine carcinoma of the cervix had exhibited tumor infiltration throughout the entire thickness of the cervix, with vascular and nerve invasion, which was a high-risk factor. Given the patient’s sensitivity to the TC regimen, a standard TC chemotherapy regimen was administered for six cycles. Additionally, considering the patient’s positive immunohistochemistry for EGFR, treatment was combined with the anti-angiogenic drug, bevacizumab.

## 3 Discussion

### 3.1 Clinical characteristics of cervical LCNEC

Large Cell Neuroendocrine Carcinoma (LCNEC) of the cervix is a rare type of cervical malignancy, accounting for less than 0.2% of all cervical cancers (1), with a median age of onset of around 36 years (2). Its signs and clinical presentations are not specific compared to common types of cervical malignancies, but cervical LCNEC is highly invasive, has a poor prognosis, and is prone to early metastasis through blood or lymphatic pathways. Additionally, cervical LCNEC possesses neuroendocrine functions, but most of the hormones secreted are inert, with only a minority of patients exhibiting corresponding neuroendocrine symptoms (such as hypoglycemia, Cushing’s syndrome, muscle weakness, carcinoid syndrome, etc (3, 4). This could be due to insufficient tumor hormone secretion or rapid metabolism in the blood, rendering them inactive and insufficient to cause corresponding symptoms. In this case, the patient had presented with abnormal vaginal bleeding, cervical occupation, and contact bleeding as the main symptoms, showing no significant difference from the clinical presentations of common cervical cancer. The progression from onset to diagnosis of advanced cervical cancer took just over a month, indicating the aggressive nature and rapid progression of the disease. Additionally, upon admission, the patient had significant abdominal distension and extensive ascites in the pelvic and abdominal cavity, suggesting pelvic implantation metastasis and not ruling out the concurrent possibility of ovarian cancer, indicating malignant ascites. However, repeated paracentesis did not reveal cancer cells, and postoperative pathology confirmed the presence of a fibroma in the right ovary (**Figure 1B**). Given the neuroendocrine function of cervical LCNEC, it cannot be ruled out that the extensive ascites were caused by hormones secreted by the tumor. At the time of consultation, the cervical lesion was visible to the naked eye, and a cervical tissue biopsy was performed according to the “three-step” diagnostic and treatment standard for cervical cancer, without cytological testing. However, postoperative immunohistochemical staining indicated P16 (+), with positive P16 staining indicating persistent infection by the Human Papillomavirus (HPV), suggesting an HPV virus infection in this patient. Similar to the common causes of cervical cancer, the occurrence of cervical LCNEC may be associated with persistent high-risk HPV16 and HPV18 infections (5, 6), indicating that the widespread use of the HPV vaccine could prevent this disease.

### 3.2 Diagnosis of cervical LCNEC

According to the 5th edition of the WHO Classification of Female Reproductive Organ Tumors published in 2020, cervical LCNEC is classified as a well-differentiated neuroendocrine tumor (7). The diagnosis of cervical LCNEC, similar to common types of cervical cancer, allows for staging based on imaging changes (FIGO 2018 edition), with PET-CT and MRI examinations providing more accurate staging (8). On H&E-stained sections (**Figure 1E**), the cytological features of cervical LCNEC could be observed: cells with abundant cytoplasm, prominent nucleoli, nuclear pleomorphism, deep staining, coarse chromatin, with tumor cells either scattered individually or clustering to form pseudorosette patterns. However, for a definitive diagnosis of cervical LCNEC, it is also necessary to demonstrate positive expression of two or more neuroendocrine-specific immunohistochemical markers, such as synaptophysin (Syn), chromogranin A (CgA), neural cell adhesion molecule (CD56), neuron-specific enolase (Nse), insulinoma-associated protein (INSM1), and vascular endothelial growth factor (VEGF) (9). In this case, the neuroendocrine markers CD56 (**Figure 1F**) and Syn (**Figure 1G**) were both positive, combined with histopathology confirming the diagnosis of large cell neuroendocrine carcinoma of the cervix. This tumor may originate from multipotent stem cells (reserve cells) in the basal layer of the cervical epithelium (10), allowing the tumor to differentiate into common types of cervical adenocarcinoma or squamous cell carcinoma, or coexist with them. Therefore, relying solely on histopathology can lead to misdiagnosis or missed diagnosis of cervical LCNEC. The discrepancy between the preoperative and postoperative pathological diagnoses in this case could be attributed to the following possible reasons:①Given the ability of cervical LCNEC to differentiate into other types of cervical cancer or coexist with them, it’s possible that neuroendocrine carcinoma and squamous carcinoma coexisted in this patient. The limited biopsy material from the cervical biopsy and the lack of immunohistochemistry could have led to a missed diagnosis. ②Cervical LCNEC could result from the transformation of cervical squamous carcinoma following neoadjuvant chemotherapy treatment. That is, cervical LCNEC originates from cervical squamous carcinoma, which survives and proliferates after treatment, or cervical LCNEC and cervical squamous carcinoma may share a common precursor cell, with cervical squamous carcinoma transforming into cervical LCNEC under the influence of neoadjuvant chemotherapy. There are individual case reports of lung adenocarcinoma patients with EGFR gene mutations transforming into small cell lung cancer pathology after treatment with tyrosine kinase inhibitors. Since this patient did not undergo related genetic testing, no evidence could be provided. ③Cervical LCNEC might have been a new tumor that emerged after neoadjuvant chemotherapy. Since cervical LCNEC may originate from reserve cells beneath the cervical epithelium, and cervical squamous carcinoma often develops from cervical intraepithelial neoplasia (CIN), which in turn originates from squamous epithelial basal cells. The postoperative pathology confirmed primary cervical LCNEC with high-grade squamous intraepithelial lesion (HSIL/CIN III), this possibility cannot be ruled out.

### 3.3 Individualized treatment for cervical LCNEC

Although there had been a pathological type transformation in this patient during treatment, the treatment was primarily aimed at addressing cervical LCNEC. Due to its rarity, there is a lack of prospective research on the treatment of cervical LCNEC, and currently, there are no unified standards. Treatment strategies often reference those used for common cervical cancer and small cell neuroendocrine carcinoma of the cervix, with a comprehensive individualized treatment plan formulated based on the patient’s condition. Regardless of the stage of the cervical tumor, systemic chemotherapy is recommended; the use of platinum-based drugs has been shown to improve the patient’s overall survival (OS) or progression-free survival (PFS). The choice of chemotherapy regimen is mainly based on platinum and etoposide (EP regimen), and to reduce recurrence, more than five cycles of chemotherapy are recommended (11–13).

**Figure 5:**
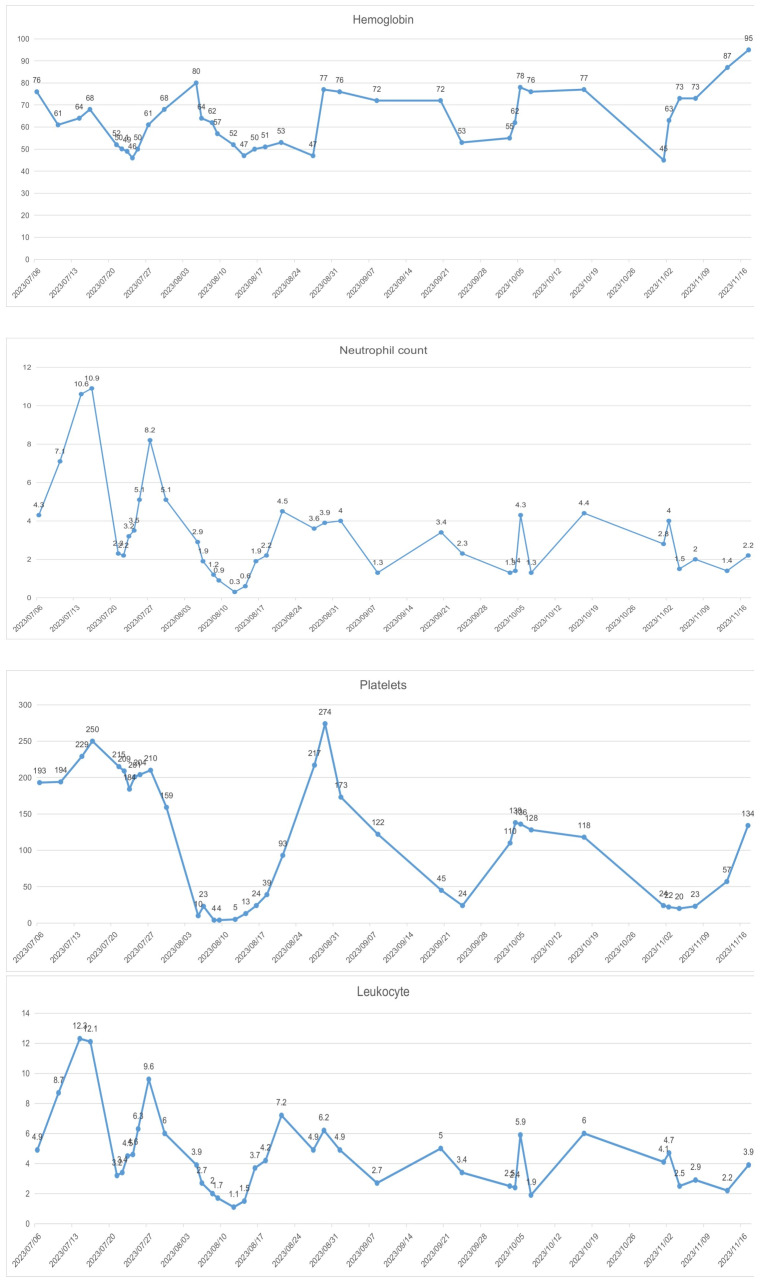
Myelosuppression.

In recent years, pelvic exenteration has gradually become one of the “salvage” treatment methods for advanced and recurrent cervical cancer. There is still controversy over whether to choose pelvic exenteration or radiochemotherapy as the initial treatment for patients with advanced cervical cancer. The NCCN guidelines recommend pelvic exenteration as the initial treatment for patients who are not suitable candidates for pelvic radiotherapy. However, for patients who are evaluated without surgical contraindications and can achieve a no visible residual R0 resection, this can significantly improve the disease-free survival rate and overall survival rate (14), with a 3-year survival rate reaching up to 73% (15), a 5-year survival rate up to 60% (16), and a 10-year survival rate up to 57% (17), even offering a potential cure. A retrospective study reported that tumors between 2 and 6 cm have a better response to neoadjuvant chemotherapy (NACT) than tumors > 6 cm, and lymphoma vascular space invasion absence was an independent prognostic factor for lymph node response to NACT. NACT followed by surgery cannot be considered a standard of care in patients with locally advanced cervical cancer(LACC), particularly in the subgroup with pre-NACT imaging suspected for lymph node metastases (18). However, Some studies have reported that, compared to the concurrent chemo-radiotherapy (CCRT) group, the neoadjuvant chemotherapy before radical surgery (NACT + RS) group may lead to improved OS(93.8% vs 56.5%) and disease free survival (DFS) (77.4% vs 33.4%) outcomes. The delayed toxicities were observed significantly more in the CCRT group compared with the NACT + RS group (19). NACT followed by radical hysterectomy has been considered an interesting alternative to CCRT for patients with LACC (20). In this case, the patient’s cervical tumor was larger than 4cm in diameter, had invaded the bladder and vagina, and there was evidence of distant metastasis, making an R0 resection unattainable upon initial evaluation. To reduce the tumor diameter, improve tumor resectability, control distant metastasis, and increase the success rate of surgery, after MDT discussion, the patient underwent a comprehensive treatment combining neoadjuvant chemotherapy + surgery + chemotherapy. For the selection of the neoadjuvant chemotherapy regimen, considering the possibility of concurrent ovarian cancer in this patient, we administered 4 cycles of *TC* chemotherapy as neoadjuvant treatment. Although the patient experienced severe myelosuppression (**Figure 5**), mainly manifested as chemotherapy-induced anemia difficult to correct, with reductions in platelets and granulocytes after chemotherapy, these issues were alleviated with active treatment and prevention, allowing the completion of subsequent chemotherapy. Moreover, the patient had developed persistent vesicovaginal urinary leakage prior to the third cycle of chemotherapy. Upon reevaluation, the tumor markers had significantly decreased compared to before chemotherapy, suggesting a notable response to the treatment regimen and control over the lesions invading the bladder. After four cycles of chemotherapy, follow-up imaging indicated that the cervical lesion had essentially disappeared, enlarged pelvic lymph nodes had reduced, and tumor markers were near normal; thus, the patient was sensitive to the *TC* chemotherapy regimen, achieving complete remission (CR) visually, which provided an opportunity for subsequent surgical treatment.

However. Multimodal treatments are associated with side effects that can impair quality of life. A multicenter study showed that LACC patients who underwent multimodal treatments seem to have a relevant impact on pelvic organ function. In particular, urinary frequency(>8 times/day) was observed significantly more in exclusive radio–chemotherapy(ERT/CT) compared with the neoadjuvant radio–chemotherapy(NART/CT)group (57.1% vs. 28.6%), as well as comparing (ERT/CT)with(NACT)(57.1% vs.17.6%). Regarding the use of sanitary pads for urinary leakage, They observed a significant difference between ERT/CT and NART/CT (42.9% vs. 14.3%)and between ERT/CT and NACT (42.9% vs. 11.8%) (21). Given the patient’s inability to tolerate continuous vaginal urinary leakage, she requested surgical treatment. Considering that the tumor lesions had essentially disappeared after neoadjuvant chemotherapy, the tumor did not involve the pelvic wall, there was no extra-pelvic lymph node metastasis, and no apparent surgical contraindications were observed. A comprehensive exploration during surgery confirmed the invasion of the cervical lesion into the bladder, urethra, and upper segment of the vagina, with enlargement of the right ovary. Thus, anterior pelvic exenteration was performed. The spleen was preserved as no cancer invasion was observed, and all other visibly affected areas were removed. An ileal conduit urinary diversion was chosen for urinary system reconstruction in this patient. To improve postoperative quality of life and prognosis and to prevent empty pelvis syndrome and poor healing of the vaginal stump, a rectus abdominis muscle flap was selected for pelvic floor reconstruction. Postoperative examination of the specimen revealed visible tumor lesions near the urethra close to the bladder and on the posterior wall of the bladder, with a leak site near the anterior urethra in the bladder trigone area (**Figure 4D**), which was the main cause of vesicovaginal urinary leakage in this patient. However, postoperatively, pathology indicated the presence of cancer in the bladder, suggesting that the tumor had disappeared after neoadjuvant chemotherapy, leading to a discrepancy between clinical and pathological diagnoses. Pathology indicated that the margins and other examined tissues were cancer-free, achieving an R0 resection. The patient recovered well postoperatively, with the ureteral stent removed and no urinary system complications observed. The stoma showed no signs of infection, the perineal wound healed well, and there was no evidence of empty pelvis syndrome.

In gynecological malignancies, disease diagnosis and prognosis are often assessed through the positive detection rates and serum level changes of tumor markers, such as the commonly used serum tumor markers SCC, HE4, CA125, etc. However, in solitary diagnosis, these can be influenced by inflammatory responses and lesions in other locations, leading to false positives. Combining these indicators can increase sensitivity and accuracy. In this case, the patient had significantly elevated levels of CA125, SCC, and HE4 before chemotherapy, which significantly decreased after chemotherapy (**Figure 3**). CA125 returned to within the normal range, but SCC and HE4 did not return to normal due to the continuous presence of the cervical lesion, right ovarian fibroma, and perisplenic abscess that had not been addressed. After surgical removal of the lesions and drainage of the abscess, the tumor markers returned to normal.

Regarding the necessity of adjuvant chemotherapy following pelvic exenteration, due to the limited research data available, there is currently no clear standard guideline. However, it is generally believed that postoperative adjuvant chemotherapy does not significantly impact the prognosis of these patients (22, 23). However, according to the NCCN guidelines for this patient with a special type of cervical cancer, where the tumor infiltrates the entire thickness of the cervix, vascular and nerve invasion are observed, and there is pelvic lymph node metastasis with the tumor size greater than 4cm, adjuvant radiochemotherapy is recommended postoperatively. Some studies have reported that, compared to the EP regimen, the TC chemotherapy regimen may lead to improved OS and progression-free survival (PFS) outcomes (24). Since this patient was sensitive to the neoadjuvant chemotherapy regimen, she continued with the standard TC regimen for adjuvant chemotherapy postoperatively. While studies have shown that for patients with locally advanced and advanced stage disease, receiving external beam radiotherapy combined with brachytherapy can improve median survival time and overall survival rates, there is currently a lack of substantial research evidence evaluating the role of adjuvant radiotherapy following pelvic exenteration (25, 26). In future treatment plans, the combination of radiotherapy will be considered.

### 3.4 Combination of targeted therapy and immunotherapy for cervical LCNEC

Patients diagnosed with advanced stage cervical LCNEC have traditionally faced a poor prognosis and high rates of disease recurrence. Incorporating targeted therapy and immunotherapy approaches may potentially offer more clinical benefits for this patient population. However, cervical LCNEC is rare, and most research on targeted therapy is still in the stage of identifying mutation targets, lacking large-scale clinical studies and a standard treatment model. In the case presented here, the patient did not undergo comprehensive molecular diagnostic testing. Given the immunohistochemical demonstration of EGFR positivity (**Figure 1H**), and the results from the NeCTuR study on the treatment efficacy for recurrent cervical neuroendocrine carcinoma, which showed that the TPB regimen (topotecan, paclitaxel, bevacizumab) could improve PFS (8.7 months vs. 3.7 months; *P*<0.0001), but not OS (16.8 months vs 14.0 months; *P*=0.49) (27), we incorporated bevacizumab into the TC regimen for combined treatment. Additionally, immune checkpoint inhibitors have shown some effectiveness in treating cervical neuroendocrine cancer, particularly in patients with positive PD-L1 expression, where the treatment effect is significant (28). The NCCN guidelines also recommend immunotherapy for patients with positive PD-L1 expression, but this patient did not undergo genetic testing, so no clinical evidence was provided. In subsequent treatments, we will actively encourage the patient to complete molecular testing to provide a basis for future immunotherapy and targeted therapy.

## 4 Conclusion

There is a pressing need to enhance our understanding and clinical awareness of cervical LCNEC, given the heterogeneous nature of this tumor type. Clinicians must remain vigilant for potential pathological transformations or underlying genetic alterations that may occur during the course of treatment. For such cases, it is essential to utilize multiple biopsy sites in conjunction with immunohistochemistry for diagnosis, and to consider re-biopsy in cases where treatment effects are not significant, to reduce the rates of misdiagnosis and missed diagnosis. This approach is of profound significance for the diagnosis and treatment of this type of disease. The case presented here is rare, and the individualized treatment plan we formulated was effectively implemented. Although radiotherapy was not concurrently administered, the patient’s general condition remains good without recurrence three months post-surgery. We will continue to follow up, as this is just a single case and large-scale clinical studies are still required for confirmation. Moreover, identifying patient-specific genetic variations and adjusting treatment plans accordingly is essential for achieving truly personalized and precise treatment.

## Data availability statement

The original contributions presented in the study are included in the article/supplementary material. Further inquiries can be directed to the corresponding author.

## Ethics statement

Ethical review and approval was not required for the study on human participants in accordance with the local legislation and institutional requirements. Written informed consent from the patients/participants or patients/participants’ legal guardian/next of kin was not required to participate in this study in accordance with the national legislation and the institutional requirements. Written informed consent was obtained from the individual(s) for the publication of any potentially identifiable images or data included in this article.

## Author contributions

CL: Writing – original draft, Data curation. MW: Writing – review & editing, Data curation, Software. WZ: Writing – review & editing, Software. XJ: Data curation, Writing – review & editing. LZ: Writing – review & editing, Formal analysis, Data curation. GW: Data curation, Formal analysis, Writing – review & editing. LH: Formal analysis, Funding acquisition, Software, Writing – review & editing.
